# A Rare Case of an Advanced Gastric Adenocarcinoma in a Teenage Syrian Boy

**DOI:** 10.1155/2018/9140593

**Published:** 2018-11-14

**Authors:** Baraa Daboul, Ahmad Ghazal, Ayham Al Halak, Sabah Hayed, Yasmin Najjar

**Affiliations:** ^1^Faculty of Medicine, University of Aleppo, Aleppo, Syria; ^2^Department of Surgery, Aleppo University Hospital, University of Aleppo, Aleppo, Syria

## Abstract

In this report, we describe a case of a gastric cancer in young age group with delayed diagnosis and poor prognosis. We report a rare case of a 16-year-old teenager with an advanced moderately differentiated gastric adenocarcinoma without any relevant history. He presented mainly with dysphagia, postprandial vomiting, and eventually hematemesis. On exploratory laparotomy, the tumor was in advanced stage, the excision was not performed, multiple biopsies and a feeding jejunostomy were done, and the patient was referred to receive a palliative therapy. Reporting such cases introduces a better understanding of the relation between gastric cancer and young ages.

## 1. Introduction

Almost one million new cases of stomach cancer were estimated to have occurred in 2012 (952,000 cases, 6.8% of the total), making it the fifth most common malignancy in the world, after cancers of the lung, breast, colorectum, and prostate [1].

Gastric carcinoma occurs most frequently in the age group of 50–70 years [2], and it is extremely rare below the age of 30; it increases rapidly and steadily to reach the highest rates in the oldest age groups [3]. Mainly, there are two subtypes of adenocarcinoma that rise from the gastric mucosa: intestinal and diffuse based on Lauren's criteria (1965). However, the 2010 WHO classification recognizes four major histologic patterns of gastric cancers: tubular, papillary, mucinous, and poorly cohesive (including signet ring cell carcinoma), plus uncommon histologic variants as discussed by Hu et al. [4].

Intestinal types are more common in males and older adults, whereas diffuse types may occur in all age groups with equal sex distribution and show more rapid progression and poorer prognosis [5].

The prognosis for young patients remains controversial. Clinicopathological features of gastric cancer are reported to differ between younger and older patients, and it is thought that the prognosis of the disease is worse for younger patients due to delayed diagnosis and more aggressive tumor behaviour as discussed by Isobe et al. [6].

## 2. Case Presentation

A 16-year-old nonsmoker teenager was admitted to the outpatient clinic complaining of a 14-month history of postprandial vomiting that progressed into hematemesis the last week. The patient was suffering from fatigue, dysphagia related to solid food, and loss of appetite which led to weight loss; the body mass index (BMI) dropped from 27.7 kg/m^2^ to 16.3 kg/m^2^ during this period; before that, the patient had been seeing many clinics outside the country without any conclusive diagnosis.

Clinical examination revealed a pale-colored skin with mild jaundice, and the abdomen did not show any palpable mass (hepatomegaly, splenomegaly, and enlarged lymph nodes), tenderness, or rebound tenderness. The remainder of the physical examination was unremarkable.

**Figure 1:**
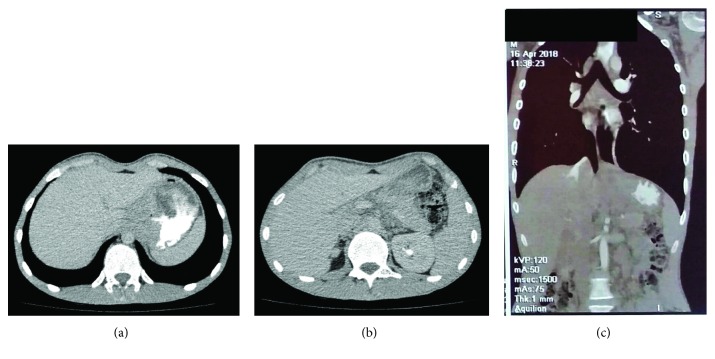
CT scan series with a radiocontrast agent showing a mass in the level of the stomach fundus invading the surrounding fatty tissue between the liver and the stomach without any liver metastasis. (a), (b) Axial sections of the abdomen. (c) Coronal section of the chest and the abdomen.

A lower esophageal sphincter narrowing was found by an upper gastrointestinal endoscopy (UGE) corresponding with a fragile bleeding gastric mass; that prevented from taking a biopsy. CT studies supported these findings by determining a large gastric mass in the level of the fundus sized 8 cm in greatest diameter, which invaded the surrounding abdominal structure (the abdominal aorta, epigastric, and hilar lymph nodes) (see Figure 1).

Laboratory studies revealed a slight elevation in both urea [54 mg/dl] and bilirubin [total 1.86 mg/dl, direct 0.58 mg/dl]. Haemoglobin values were slightly decreased [10.4 g/dl], and carcinoembryonic antigen (CEA) value was within the normal ranges. The patient did not have any relevant medical or familial history.

For closer investigation, the patient underwent an exploratory laparotomy which revealed peritoneal implantations in addition to that mass; which was left in the place without excision, several biopsies were taken from the mass and the regional lymph nodes (the retroperitoneum, mesenteric nodes); and finally, a feeding jejunostomy was done.

**Figure 2:**
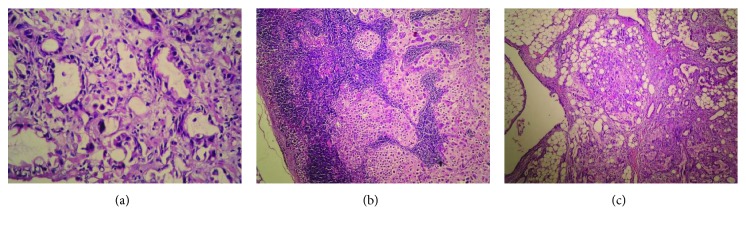
Histologic examination of the resected specimens with hematoxylin and eosin revealing moderately differentiated adenocarcinoma. (a) From the stomach (magnification ×40), it shows the hypercellularity with basophilic, oligocytoplasmic, and pleomorphic cells and relatively large nuclei with dense chromatin and loose desmoplastic stroma which contains lymphocytic and plasmocytic infiltration. (b) From the resected lymph nodes (magnification ×10), it shows the islets of metastatic infiltration of the tumor cells into the nodes; moderate. (c) From the omentum (magnification ×10), it shows the metastatic infiltrative cells forming multisized islets.

Pathology studies reported a moderately differentiated adenocarcinoma of the stomach grade II with lymph node metastasis and peritoneal carcinomatosis (see Figure 2). The tumor infiltrates into the surrounding structures (T4b), there is metastasis in about 9 lymph nodes (N3), and there is no distant metastasis (M0) as shown in CT studies; these findings give stage IIIC to the tumor according to the American Joint Committee on Cancer (AJCC) 7th edition.

The patient was referred to the oncologic department where a PET scan was ordered to set him on chemotherapy, and he died 4 months after surgery.

## 3. Discussion

Gastric carcinoma occurs most frequently in the age group of 50–70 years [2], and it is extremely rare below the age of 30 [3]. However, the rate of gastric cancer in young patients has increased over the past few decades, despite a reduction in the overall prevalence of the disease as mentioned by Pisanu et al. [7]. For that reason, reporting such cases introduces a better understanding of the relation between gastric cancer and young ages.

Epidemiological studies in different populations show that the most consistent association is diet (such as smoked meat, salty foods, and chili peppers); this is especially true of intestinal-type carcinomas, whereas adequate intake of fresh fruits and vegetables lowers the risk. Additional risk factors include smoking, helicobacter pylori infection, pernicious anemia, and prolonged use of proton-pump inhibitors (PPIs). Familial associations were described with familial adenomatous polyposis (FAP) and hereditary nonpolyposis colorectal cancer (HNPCC—Lynch syndrome II), and none of them seems to be presented in our case according to the patient's history. Although helicobacter pylori infection is a strong risk factor, it was not tested in our patient who might be positive for H. pylori.

The presence of gastrointestinal symptoms in the young patients may mislead the physicians in the diagnostic process, as what happened in our patient case, who presented primarily with dysphagia, postprandial vomiting, and later with fatigue, loss of weight, and hematemesis. These initial symptoms in this group of age may not refer to gastric cancer, and hence, that may delay the diagnosis, where the invasion included the peritoneum, abdominal aorta, and regional lymph nodes, which prevented us from a curative resection, and eventually a poor prognosis.

In gastric cancer, the sensitivity of CEA is of approximately 30% [8], and increased preoperative CEA level signifies tumor invasion into the serosa of the stomach [9]. In our case, CEA values were within normal ranges, which makes it difficult to rely on them as a predictive factor. These normal values with the unremarkable physical examination in this patient (no palpable abdominal mass or lymph nodes) also could be a cofactor in delaying the diagnosis.

In fact, these multiple factors do not exempt doctors from taking responsibilities in delaying the diagnosis, which is not good, as time here is paramount in determining the management and the prognosis. This could be attributed to not following a proper academic approach while investigating him, because a period of 14 months with recurrent visits for doctors (outside the country) is more than enough to set a diagnosis.

Thus, the clinicians should be academic in investigating the patients, especially when recognizing red flag findings related to gastrointestinal tract such as hematemesis and unexplained weight loss in this patient.

## 4. Conclusion

We conclude the case with the importance of including the gastric tumor in the differential diagnosis in every patient regardless of her/his age whenever symptoms of dysphagia, loss of appetite, and hematemesis present, and further investigations should be done, as the early diagnosis of a gastric tumor in this group plays a crucial role in determining the prognosis.
